# Activation of JNK1/2 and p38 MAPK signaling pathways promotes enterovirus 71 infection in immature dendritic cells

**DOI:** 10.1186/1471-2180-14-147

**Published:** 2014-06-07

**Authors:** Hongjun Peng, Mei Shi, Li Zhang, Yuanyuan Li, Jing Sun, Lirong Zhang, Xiaohui Wang, Xiaopeng Xu, Xiaolei Zhang, Yijie Mao, Yun Ji, Jingting Jiang, Weifeng Shi

**Affiliations:** 1 Department of Clinical Laboratory, the Third Affiliated Hospital of Suzhou University, No. 185 Juqian street, Changzhou, Jiangsu 213003, P. R. China; 2 Department of Oncology Laboratory, the Third Affiliated Hospital of Suzhou University, Changzhou, Jiangsu 213003, China

**Keywords:** Enterovirus 71, Viral replication, JNK1/2, p38 MAPK, Dendritic cells

## Abstract

**Background:**

c-Jun NH_2_-terminal kinase/stress-activated kinase (JNK/SAPK) and the p38 mitogen-activated protein kinase (p38 MAPK) are important components of cellular signal transduction pathways, which have been reported to be involved in viral replication. However, little is known about JNK1/2 and p38 MAPK signaling pathways in enterovirus 71 (EV71)-infected immature dendritic cells (iDCs). Thus, iDCs were induced from peripheral blood mononuclear cells (PBMC) and performed to explore the expressions and phosphorylation of molecules in the two signaling pathways as well as secretions of inflammatory cytokines and interferons during EV71 replication.

**Results:**

We showed that EV71 infection could activate both JNK1/2 and p38 MAPK in iDCs and phosphorylate their downstream transcription factors c-Fos and c-Jun, which further promoted the production of IL-2, IL-6, IL-10, and TNF-α. Moreover, EV71 infection also increased the release of IFN-β and IL-12 p40. Pretreatment of iDCs with SP600125 and SB203580 (20 μM) could severely impair viral replication and its induced phosphorylation of JNK1/2,p38 MAPK, c-Fos and c-Jun. In addition, treatment of EV71-infected iDCs with SP600125 and SB203580 could inhibit secretions of IL-6, IL-10 and TNF-α.

**Conclusion:**

JNK1/2 and p38 MAPK signaling pathways are beneficial to EV71 infection and positively regulate secretions of inflammatory cytokines in iDCs.

## Background

EV71 is a positive-stranded RNA virus in the genus enterovirus of the family Picornaviridae, usually leading to hand, foot, and mouth diseases (HFMD) and herpangina [1,2]. Moreover, EV71 has also been associated with fatal pulmonary edema, severe neurological complications, including encephalitis, meningitis, and a poliomyelitis-like syndrome [3,4]. Increasing evidences have found it to be the major etiological agent causing current outbreaks of HFMD in the Asia-Pacific region, including mainland China [2,5,6]. However, the molecular pathogenesis of EV71 infection remains obscure.

Mitogen-activated protein kinase (MAPK) belongs to a family of serine/threonine protein kinases. It is widely conserved among eukaryotes and involved in many cellular processes such as inflammation, proliferation, differentiation, movement, and death [7-9]. To date, seven distinct groups of MAPKs have been characterized in mammalian cells, including extracellular regulated kinases (ERK1/2), JNK1/2/3, p38 MAPK (p38 α/β/γ/δ), ERK3/4, ERK5, ERK7/8 and Nemo-like kinase (NLK) [10-12]. Of these, the most extensive studies are ERK1/2, JNKs and p38 MAPKs. As previously reported, JNK1/2 and/or p38 MAPK pathways is required for infection and replication of human immunodeficiency virus type 1, encephalomyocarditis virus, coxsackievirus B3, hepatitis C virus, herpes simplex virus 1, and the severe acute respiratory syndrome coronavirus [13-18]. The diverse effects of JNK1/2 and p38 MAPK activation by these viruses include induction of apoptosis in infected cells and enhancement of viral replication.

DCs are the first line of defense which could not only promote innate immune response but also initiate specific host immune responses by both capturing and processing antigens to MHC-I and II molecules on the cellular surface, regulating naïve T cells and differentiation [19-21]. It has been reported that JNK1/2 and p38 MAPK signal cascades are required for EV71 replication in rhabdomyosarcoma (RD) cells and SK-N-SH cells [22-24]. However, little is known about the roles of JNK1/2 and p38 MAPK signaling pathways in DCs during the course of EV71 infection. In the present study, iDCs were induced from PBMC isolated from healthy blood donors in the presence of granulocyte-macrophage colony-stimulating factor (GM-CSF) and IL-4, which used to investigate the expressions and phosphorylation of molecules in JNK1/2 and p38 MAPK signaling pathways as well as secretions of inflammatory cytokines and interferons during EV71 replication.

## Methods

### Ethics statement

All the patients provided informed consents, which was approved by the Ethics Committee of the Third Affiliated Hospital of Suzhou University.

### Antibodies and chemicals

Dulbecco’s modified Eagle’s medium (DMEM), fetal bovine serum (FBS) and RPMI 1640 were purchased from Thermo Scientific HyClone (UT, USA). Hybond C membrane and ECL Western blot detection system were from Pierce (Rockford, IL, USA). Rabbit polyclonal antibodies against JNK, p-JNK, p38 MAPK, p-p38 MAPK, c-Fos, p-c-Fos, c-Jun, p-c-Jun and horseradish peroxidase (HRP) conjugated goat anti-rabbit IgG were purchased from SAB (Pearland, TX, USA). Antibodies against anti-glyceraldehyde-3-phosphate dehydrogenase (GAPDH) were obtained from ProteinTECH Group (Chicago, IL, USA). Rabbit polyclonal antibody against EV71 VP1 was purchased from Abcam (Cambridge, UK). The JNK1/2 and p38 MAPK specific inhibitor (SP600125 and SB203580) were acquired from LC Laboratories (Woburn, MA, USA) and freshly prepared using DMSO solution.

### Cell culture and virus propagation

RD cells were purchased from Chinese Academy of Sciences Cell Bank of Type Culture Collection (CBTCCCAS), cultured in high glucose DMEM supplemented with 10% fetal bovine serum (Gibco, CA, USA) at 37°C in a humidified incubator under 5% CO_2_ atmosphere, and passaged when reaching 90% confluence. EV71 strain was from China Center for Type Culture Collection (CCTCC)/GDV083 (ATCC VR-784) and propagated in RD cells. Viral titer was determined by CPE and expressed as 50% tissue culture infective dose (TCID50) per ml [25].

### Generation of DCs

Peripheral venous blood obtained from healthy blood donors was kindly provided by Changzhou Blood Center and used to purify mononuclear cells using Ficoll-Hypaque (Invitrogen, CA, USA) density gradient centrifugation. Monocytes were isolated from PBMC by adhesion to plastic dishes for more than 2 h at 37°C as previously described. iDCs were generated from monocytes by culturing in RPMI 1640 medium containing 10% FBS, 100 ng/mL of GM-CSF (Hainan Pharmaceutical Co., China), 50 ng/mL of IL-4 (PeproTech, NJ, USA), and antibiotics for 7 days. On Day 7, cells were collected and analyzed by flow cytometry (Beckman coulter, CA, USA) for CD3, CD11c, CD80, CD83, CD86 and HLA-DR. The induced DCs were assigned in two groups. One group was not infected with EV71 and used as control. The other group was infected with EV71 at a MOI of 5 for 1 h at 37°C. After washed twice with PBS, all cells were cultured in RPMI medium for 24 h and analyzed using flow cytometry. Meanwhile, the supernatants were collected and stored at -80°C.

### Total RNA preparation and PCR arrays

After incubating at 37°C for 1/2 h, 2 h, 8 h and 24 h, both uninfected and infected iDCs were harvested and used to extract total RNA using the SV total RNA isolation system (Promega, Madison, WI, USA). PCR arrays were performed with customized PCR containing pre-dispensed primers (CT biosciences, China) on the LightCycler 480 (Roche Diagnostics, Mannheim, Germany) using SYBR MasterMix (catalog # CTB101; CT biosciences, China). Each PCR contained 10 ng of synthesized cDNA. The thermocycler parameters were performed with an initial denaturation at 95°C for 5 min followed by 40 cycles of denaturation at 95°C for 15 s, annealing at 60°C for 15 s and extension at 72°C for 20 s. Relative change in gene expression was calculated using ΔΔCt (threshold cycle) method. The housekeeping genes such as B2M, ACTB, GAPDH, RPL27, HPRT1 and OAZ1 were used to normalize to the amount of RNA. Fold changes in gene expression were calculated using the formula of 2^-ΔΔCt^.

### Cell extraction and western blot analysis

iDCs were pre-incubated for 1 h with SP600125 and SB203580 (20 μM), and then infected with EV71 at a MOI of 5 in the presence of SP600125 and SB203580 for 24 h. Cells were harvested by centrifugation, washed and lysed with a lysis buffer (2% sodium dodecyl sulfate, 35 mM *β*-mercaptoethanol, 50 mM Tris–HCl (pH 6.8), 1 mM phenylmethylsulfonylfluoride). Cell lysates were obtained by centrifugation at 45,000 × g for 1 h at 4°C. Total protein concentration was determined by the bicinchoninic acid protein assay kit (Pierce). Equal amount of proteins were subjected to sodium dodecyl sulfate polyacrylamide gel electrophoresis (SDS-PAGE), and transferred onto PVDF membranes (Millipore). The membranes were blocked for 2 h with 5% nonfat dry milk solution in Tris-buffered saline containing 0.1% Tween-20 and then incubated with specific primary antibodies. After washed with PBS, the membranes were incubated with HRP conjugated secondary antibodies and washed with PBS. The immunoreactive bands were detected by ECL reagents (GE Healthcare), visualized on Super RX film (Fujifilm) and quantitated by densitometric analysis (ImageQuant, Molecular Dynamics and PDSI, GE Healthcare). The level of phosphoproteins was normalized to its respective control at 0 h, which was arbitrarily set to 1.

### Evaluation of cytokine levels by luminex fluorescent technique

iDCs were infected with EV71 at a MOI of 5 for 1 h at 37°C, washed twice and cultured in RPMI medium. The supernatants were collected at 24 h p.i. by centrifugation at 3, 000 × g for 30 min and used to measure the concentrations of IL-2, IL-6, IL-10, IL-12 p40, IL-12 p70, TNF-α, INF-α and IFN-β with Milliplex magentic beads (Millipore, Billerica, MA, USA) using luminex fluorescent technique according to the manufacturers’ instruction. The fluorescence data in each standard, quality control and samples were detected with the FLEXMAP3D (Luminex Co., TX, USA) and subsequently analyzed using the MILLIPLEX™ Analyst V5.1 (VigeneTech Inc, Carlisle, MA, USA). The standard curves were generated for each cytokine with Bio-plex manager software and used to calculate cytokine concentrations in supernatants using stepwise five-fold dilution of protein standards.

### Statistical analysis

All data were presented as the mean ± SE and statistically analyzed using GraphPad Prism software (San Diego, CA). P values less than 0.05 were considered statistically significant.

## Results

### Differential mRNA expressions of molecules in JNK1/2 and p38 MAPK signaling pathways

iDCs were prepared from monocytes purified from peripheral blood by induction with GM-CSF and IL-4. Flow cytometric analysis indicated that 90.8% and 92.9% of DCs were positive for CD80 and CD11c, respectively, and only 3.5% and 6.8% of cells were positive for CD3 and CD83, respectively, confirming that they were indeed iDCs. At 1/2 h, 2 h, 8 h and 24 h p.i., iDCs were collected and the expressions of molecules in JNK1/2 and p38 MAPK signaling pathways were examined by PCR arrays. The results showed that the mRNA levels of MEK3/6, MEK4/7, JNK1, JNK2, JNK3, and p38 MAPK(α/β) were upregulated by 2.02 - 3.08 - fold at different times of EV71 p.i. in different time, while c-Jun and c-Fos were increased by 3.03 to 9.17 - fold. In addition, the mRNA levels of IL-2, IL-6, IL-12, TNF-α, and IFN-β were upregulated by 2.24 - 4.32 - fold at different times of EV71 p.i. (Table 1).

**Table 1:** Differential mRNA expressions of molecules in JNK1/2 and p38 MAPK signaling pathways in EV71-infected iDCs at different time points

**Gene symbol**	**EV71/control (Fold changes)**			
	**1/2 h**	**2 h**	**8 h**	**24 h**
MAP2K3 (MEK3)	+1.58	**+3.08**	+1.13	+1.05
MAP2K4 (MEK4)	+1.25	+1.16	+**2.05**	-1.11
MAP2K6 (MEK6)	-1.08	**+2.30**	+1.76	+1.08
MAP2K7 (MEK7)	+1.61	+1.10	+**2.75**	+1.00
MAPK8 (JNK1; SAPK1)	+1.27	+**2.30**	+1.10	+1.40
MAPK9 (JNK2; SAPK)	+1.14	+1.31	+**2.59**	+1.18
MAPK10 (JNK3)	+1.89	+1.94	+**2.51**	-1.80
MAPK11 (p38-β MAPK)	+1.10	+**2.81**	+1.72	+1.01
MAPK12 (p38–γ MAPK)	+1.28	+1.06	+1.76	+1.25
MAPK13 (p38 -δ MAPK)	+1.39	-1.54	-1.15	-1.01
MAPK14 (p38-α MAPK)	+1.36	+1.30	+**2.02**	+1.19
c-Jun	+1.28	+1.89	+**3.03**	+**3.30**
c-Fos	+**9.17**	+**8.12**	+**4.05**	+**3.32**
IFN-α1	-1.04	+1.79	+1.24	**-2.15**
IFN-β	-1.10	**+2.24**	+1.68	**-2.02**
IL-2	-1.09	+**4.32**	+1.40	**-4.88**
IL-6	-1.27	+**2.83**	-1.73	-1.25
IL-10	-1.06	+1.91	+1.14	+1.18
IL-12	+1.01	+1.22	+**2.67**	+1.49
TNF-α	+1.59	+**2.44**	+1.45	+1.74

Upregulated and downregulated transcripts are indicated as ‘+’ and ‘–’ values, respectively. The changes of mRNA expressions (≥2 or ≤-2 – fold) are indicated by boldface letters.

#### EV71 replication was inhibited in iDC preincubated with SP600125 and SB203580

**Figure 1:**
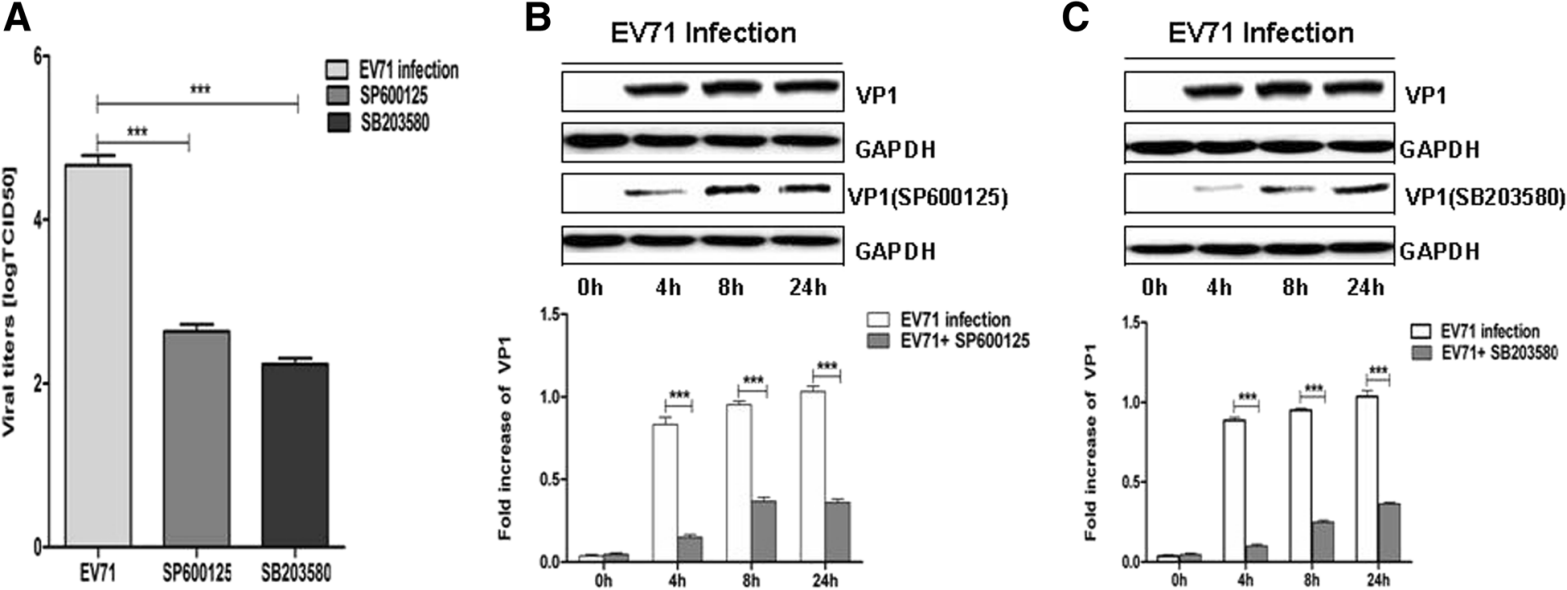
**The inhibitory effect of SP600125 and SB203580 on EV71 replication. (A)** iDCs (3 × 10^5^/well) pretreated with or without SP600125 and SB203580 (20 μM) for 1 h and infected with EV71 (MOI = 5) for 24 h, and culture supernatants were collected after infection to determine viral titers. **(B and C)** Western blot results of the supernatants and cell lysates of iDCs pre-incubated without or with SP600125 and SB203580 (20 μM) for 1 h and infected with EV71 (MOI = 5), using a specific antibody against VP1. The intensity of VP1 protein band quantitated by densitometric analysis and normalized to GAPDH. The data were expressed as mean ± SE from three independent experiments and analyzed by two-way ANOVA (***p < 0.001).

As specific inhibitors of JNK and p38 MAPK, SP600125 and SB203580, respectively, were used to study the effects of JNK1/2 and p38 MAPK activation on EV71 replication. iDCs pre-incubated with or without SP600125 and SB203580 (20 μM) for 1 h and infected with EV71 at a MOI of 5 for 24 h and the repilcation of EV71 was measured by TCID50. The results showed that the two inhibitors markedly inhibited EV71 replication (Figure 1A). Meanwhile, expression of EV71 VP1 protein in iDCs treated with SP600125 and SB203580 (20 μM) significantly reduced expression of EV71 VP1 protein at 4 h, 8 h and 24 h p.i., respectively (Figure 1B and C).

### Activation of JNK1/2 and p38 MAPK during EV71 infection

**Figure 2:**
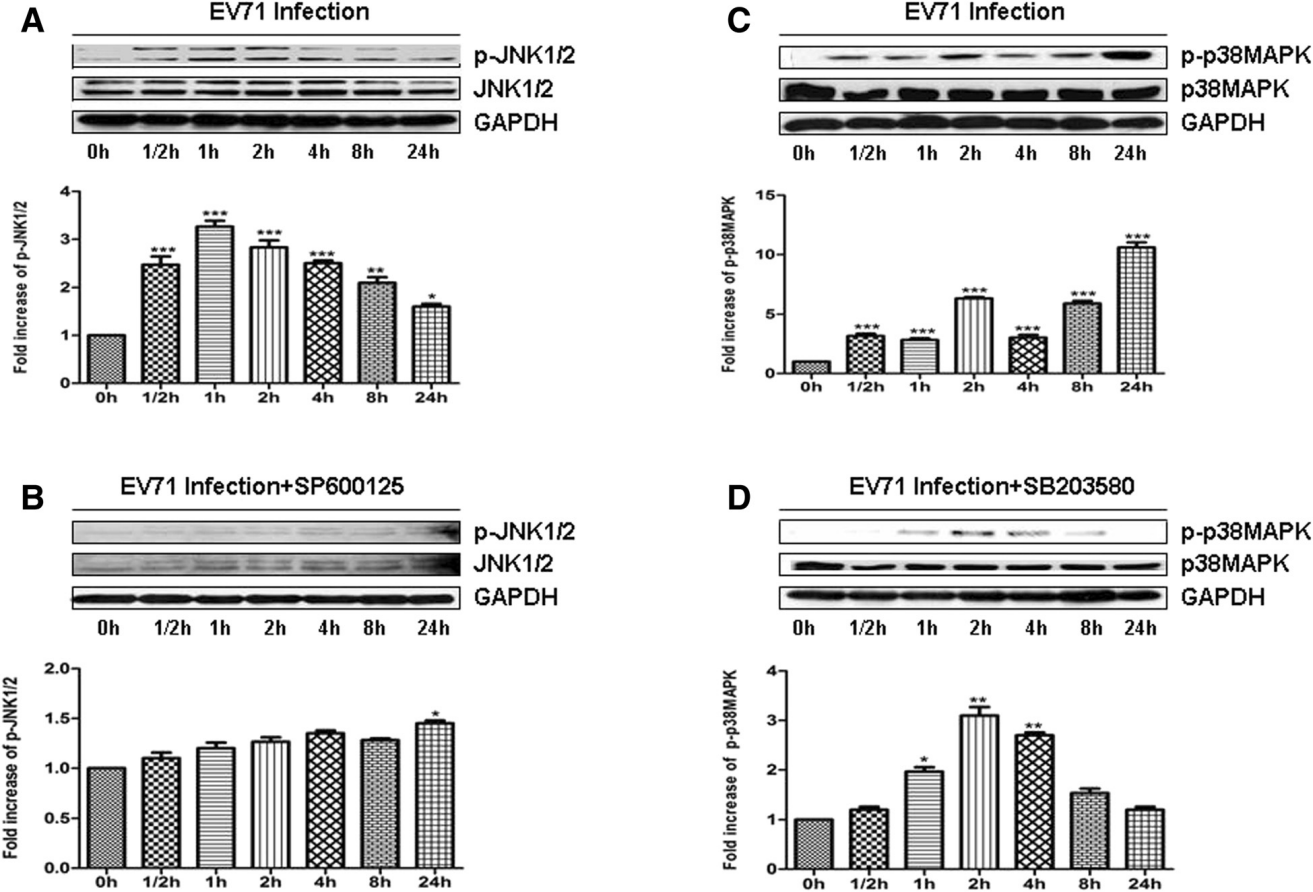
**EV71 infection stimulates activation of JNK1/2 and p38 MAPK. (A and C)** Western blot analysis of cell lysates of iDCs infected with EV71 at a MOI of 5 at 0 h, 1/2 h, 1 h, 2 h, 4 h, 8 h and 24 h p.i. using antibodies against total or phosphorylated JNK1/2, p38 MAPK, as well as internal control GAPDH. **(B and D)** Western blot analysis of cell lysates of iDCs preincubated with SP600125 and SB203580 (20 μM) for 1 h and infected with EV71 at a MOI of 5 at indicated times using antibodies against total and phosphorylated JNK1/2, p38 MAPK, as well as internal control GAPDH. The intensities of phosphorylated JNK1/2 and p38 MAPK were normalized to control level. The data were expressed as mean ± SE from three independent experiments and analyzed by one-way ANOVA (*p < 0.05, **P < 0.01 and ***P < 0.001).

It has been reported that JNK1/2 and p38 MAPK are phosphorylated during various virus infection [26,27]. In order to assess whether activation of these two MAPK signaling pathways occurred in EV71-infected iDCs, the degrees of total and phosphorylated JNK1/2 and p38 MAPK at 0 h, 1/2 h, 1 h, 2 h, 4 h, 8 h and 24 h p.i. were examined by Western blot. The results showed that EV71 infection enhanced not only mRNA levels of JNK1/2 and p38 MAPK (Table 1) but also their phosphorylation with prolonged infection. The phosphorylation of JNK1/2 reached its peak at 1 h p.i. (Figure 2A), while that of p38 MAPK reached its peak at 2 h and 24 h p.i., respectively(Figure 2C). Furthermore, the phosphorylation of JNK1/2 and p38 MAPK in EV71-infeced iDCs were significantly suppressed by pretreatment with JNK1/2 and p38 MAPK inhibitor (SP600125 or SB203580) (Figure 2B and D). Therefore, JNK1/2 and p38 MAPK play important roles in EV71 replication cycle and phosphorylation of downstream molecules.

### EV71 infection activates and phosphorylates c-Fos and c-Jun

**Figure 3:**
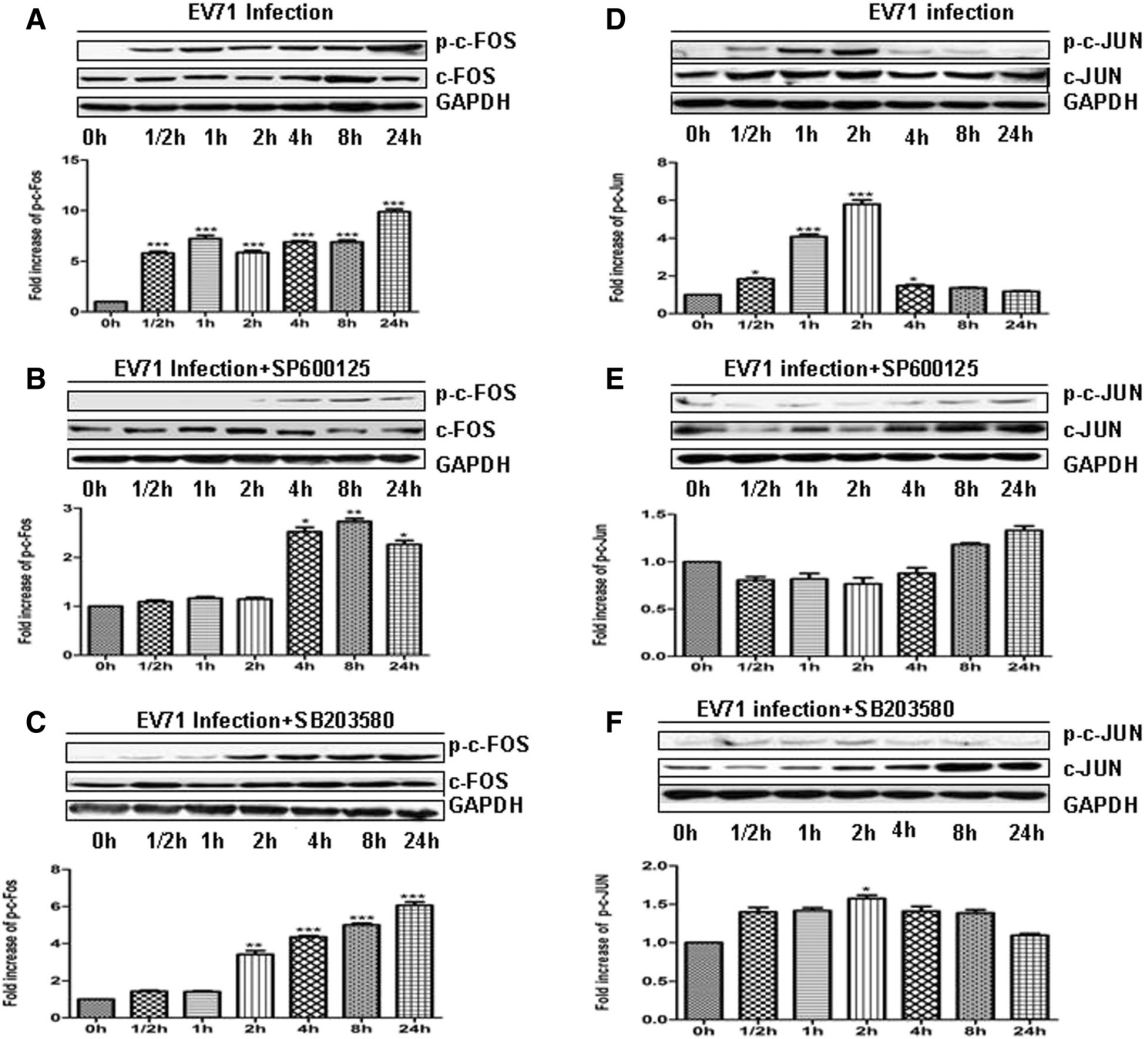
**Phosphorylation of c-Fos and c-Jun in EV71-infected iDCs. (A and D)** The western blot results of cell lysates collected at indicated times of iDCs infected with EV71 (MOI = 5) for 24 h using antibodies against total and phosphorylated c-Fos and c-Jun. **(B and E)** The western blot results of cell lysates collected at indicated times of iDCs pretreated with SP600125 (20 μM) for 1 h and infected with EV71 (MOI = 5) for 24 h using antibodies against total and phosphorylated c-Fos and c-Jun. **(C and F)** The western blot results of cell lysates collected at indicated times of iDCs pretreated with SB203580 (20 μM) for 1 h and infected with EV71 (MOI = 5) for 24 h using antibodies against total and phosphorylated c-Fos and c-Jun. The intensities of phosphorylated c-Fos and c-Jun were quantitated and normalized as described. The data were expressed as mean ± SE from three independent experiments and analyzed by one-way ANOVA (*p < 0.05, **p < 0.01, ***p < 0.001).

The activator protein 1 (AP-1) is a heterodimeric transcription factor composed of proteins in the subfamilies of c-Jun, c-Fos, Maf, and activating transcription factor (ATF). It regulates gene expression in response to a variety of stimuli, including cytokines, growth factors, stress, and bacterial and viral infections [28,29]. The results of RT-PCR showed that EV71 infection (MOI = 5) upregulated the expressions of c-Fos and c-Jun at mRNA level. To further investigate whether EV71 infection could activate and phosphorylate c-Fos and c-Jun, total and phosphorylated c-Fos and c-Jun were detected by Western blot. The results showed that c-Fos was rapidly phosphorylated by EV71 infection, reaching its peak at 24 h p.i. (Figure 3A) and this effect was inhibited by pretreatment with SP600125 for 1 h (Figure 3B), but delayed by pretreatment with SB203580 (Figure 3C). Similarly, c-Jun was also rapidly phosphorylated by EV71 infection, reaching its peak within 2 h p.i. (Figure 3D). And this effect was significantly attenuated by pretreatment with SP600125 and SB203580 (Figure 3E and F). The data demonstrate that EV71 infection triggers JNK1/2 or p38 MAPK-mediated activation of c-Fos and c-Jun.

### Secretions of IL-2, IL-6, IL-10, IL-12, TNF-α,IFN-α and IFN-β

**Figure 4:**
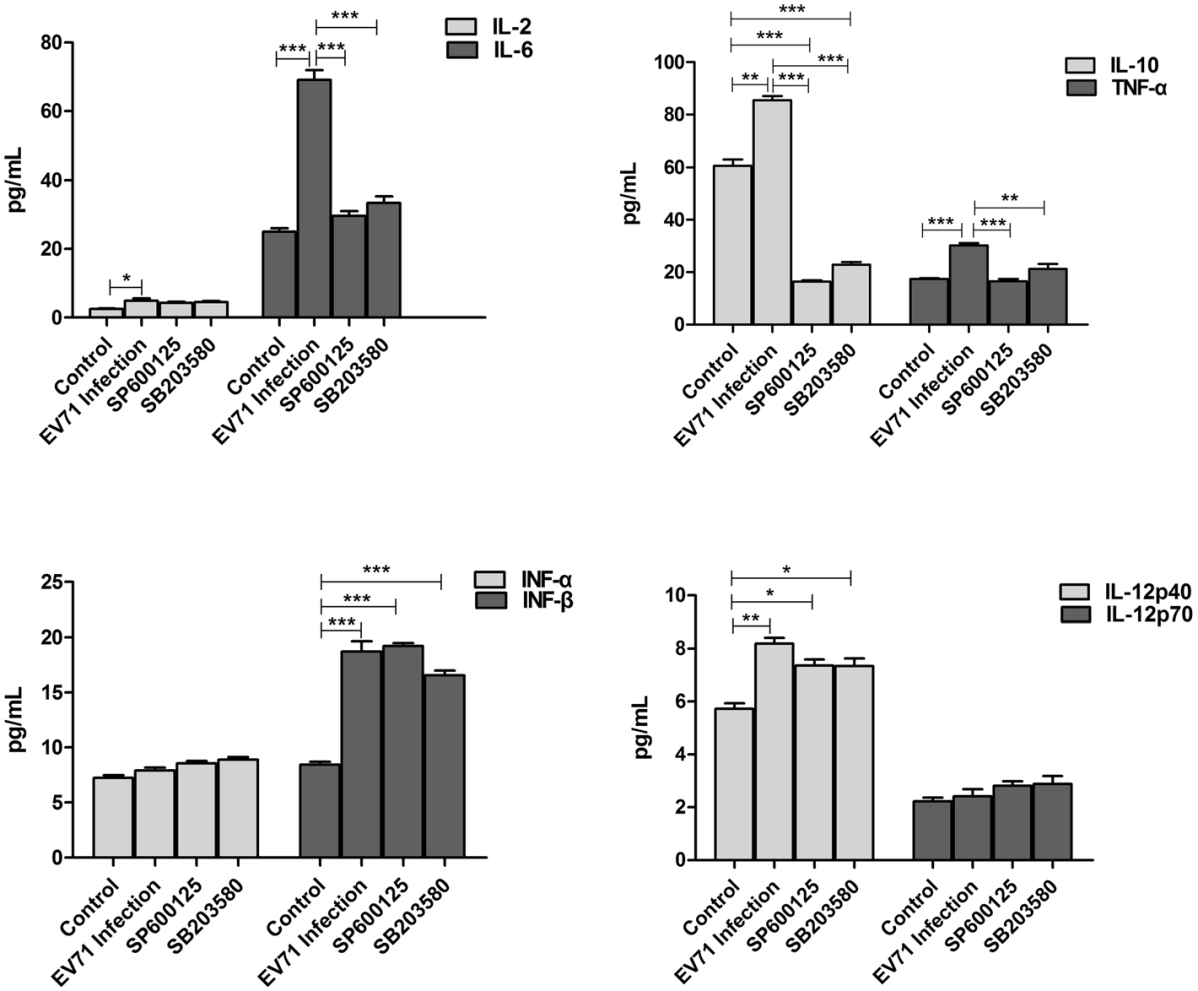
**EV71 infection enhances secretions of proinflammatory cytokines in iDCs.** Control: Uninfected iDCs; EV71 infection: EV71-infected iDCs; SP600125 or SB203580: iDCs preincubated with inhibitor SP600125 or SB203580. The cytokine levels of uninfected iDCs and EV71-infected iDCs with or without inhibitor SP600125 and SB203580 (20 μM) in the culture supernatants were harvested at 24 h p.i. were measured by luminex fluorescence technique. The data were expressed as mean ± SE from at least three independent experiments and analyzed by one-way ANOVA with Bonferroni post-hoctests (*p < 0.05, **P < 0.01 and ***P < 0.001).

iDCs can secrete several cytokines once they are activated by viral infection. To examine the role of JNK1/2 or p38 MAPK pathways in cytokine secretion in iDC, the culture supernatants of control iDCs, EV71-infected iDCs and iDCs pretreated with inhibitor SP600125 or SB203580 (20 μM) prior to EV71 infection were collected at 24 h p.i. and used to detect the levels of IL-2, IL-6, IL-10, IL-12 p40, IL-12 p70, TNF-α, IFN-α and IFN-β using luminex fluorescent technique. The results showed that EV71 infection (MOI = 5) significantly increased secretions of IL-2, IL-6, IL-10, IL-12 p40, TNF-α and IFN-β in iDCs, and pretreatment with SP600125 or SB203580 only significantly inhibited the production of IL-6, IL-10 and TNF-α, but not that of IL-2, IL-12 p40, IL-12 p70, IFN-α and IFN-β, indicating that production of the formers, but not the latters, were mediated by JNK1/2 or p38 MAPK pathways (Figure 4).

## Discussions and conclusion

EV71 is a neurotropic picornavirus. Its infection could lead to neurological manifestations, ranging from aseptic meningitis to acute flaccid paralysis and brainstem encephalitis and is often associated with systemic features, such as severe pulmonary edema and shock, in young children [4]. The pathogenesis of its adverse clinical outcomes may be related to cell tropism, cell death and host immune responses, etc. [30]. DCs are essential for the induction of innate and specific immune responses against invading pathogens [31,32]. Previous studies have shown that EV71 and dengue viruses could increase the viability, activation, cytokine release and T-cell priming activity of DCs [33,34]. Particularly, iDCs are highly specialized and efficient in uptaking and processing antigens including various viruses. Whereas, JNK1/2 and p38 MAPK signaling pathways also play important roles in proinflammatory cytokine secretions and EV71 replication [27,35]. However, whether EV71 infection could activate JNK1/2 and p38 MAPK in iDCs and the roles of their activation on EV71 replication have not been well explored. In this study, we investigated the effects and underlying mechanisms of JNK1/2 and p38 MAPK signaling pathways on EV71 infection in iDCs that are differentiated from PBMC.

The mammalian JNKs are encoded by three distinct genes (jnk1, jnk2 and jnk3), and they are strongly activated in response to cytokines, UV irradiation, growth factor deprivation, DNA damaging agents, growth factors,and viral infection [36,37]. JNK1 and JNK2 are expressed in most cell types, while JNK3 is found only in brain and testis [38]. The upstream activators for JNK pathway, i.e., MAP2Ks, are MEK4 and MEK7.The diversity of upstream activators of MEK4 and MEK7, which enable JNK pathway activation by a large number of external stimuli. In the present study, EV71 infection increased mRNA levels of MEK4, MEK7 and JNK1/2, and enhanced JNK1/2 phosphorylation with prolonged infection. The phosphorylation of JNK1/2 reached its peak at 1 h p.i. Pretreated with inhibitor SP600125 significantly suppressed the phosphorylation of JNK1/2 and EV71 propagation, indicating that EV71 infection triggered JNK1/2 pathway and phosphorylation of JNK1/2 may be essential for EV71 replication.

Four isoforms of p38 MAPK have been identified and named as p38 MAPK α/β/γ/δ [39]. Like all MAPKs, p38 MAPK kinases are activated by dual kinases MAP2Ks (e.g., MEK3 and MEK6, etc.) and several MAP3Ks, including MTK1, MLK2/MST, MLK3, ASK1 and TAK1, have been reported to cause p38 MAPK activation [40,41]. These kinases may confer the specificity of response to different stimuli including virus infection. All MAPKs, including JNK and p38 MAPK, are activated by MAPK kinases-mediated dual Thr and Tyr phosphorylation [42,43]. These residues phosphorylated during activation are Thr183/Tyr185 of JNK and Thr180/Tyr182 of p38 MAPK. In this study, EV71 infection promoted mRNA levels of MEK3, MEK6 and p38 MAPK, as well as phosphorylation of p38 MAPK. Pretreatment of EV71-infeced iDCs with p38 MAPK inhibitor SB203580 significantly inhibited the phosphorylation of p38 MAPK and EV71 replication, indicating that p38 MAPK pathway also plays an important role in EV71 infection.

The transcription factor activator protein 1 (AP-1) is a major downstream target of JNK1/2 and p38 MAPK. It is a dimeric complex composed of members of the c-Jun, c-Fos, Maf, and ATF protein subfamilies. After activation in the cytoplasm, JNK1/2 and p38 MAPK translocate to the nucleus, where they phosphorylate Ser and Thr residues of specific AP-1 subunits to augment AP-1 transcriptional activity. Both JNK1/2 and p38 MAPK target to ATF2 (ATF subfamily), while JNK1/2 also targets to c-Jun and JunD [44]. Our results showed that EV71 infection enhanced mRNA level of c-Fos and c-Jun, and rapidly induced phosphorylation of c-Fos and c-Jun within 2 h. EV71-induced c-Jun phosphorylation was completely inhibited by inhibitor SP600125 and SB203580. In addition, c-Fos phosphorylation was inhibited by SP600125, but delayed by SB203580. Thus, we speculated that JNK1/2 is the major kinase responsible for c-Fos phosphorylation. These results indicated that EV71 infection of iDC could activate JNK1/2 and p38 MAPK signaling pathway cascades, which inturn phosphorylated their downstream molecules such as c-Jun and c-Fos, and subsequently promted the secretions of proinflammatory cytokines.

Proinflammatory cytokines such as IL-6, TNF-α, and IFN-β are usually induced by oxidant stress, cytokines, and virus infection, which play important roles in host cell damages, chronic inflammation, and other immunoresponses [45-49]. EV71 infection can stimulate DCs to secrete various cytokines [33]. In the present study, EV71 infection of iDCs significantly increased the productions of IL-2, IL-6, IL-10, IL-12 p40, TNF-α and IFN-β. Moreover, the enhanced secretions of IL-6, IL-10 and TNF-α, but not IL-12 and IFN, were remarkably inhibited by pretreatment with SP600125 and SB203580, indicating that the enhanced secretions of proinflammatory cytokines, but not IL-12 and IFN, by EV71 infection were mediated by JNK1/2 and p38 MAPK signaling pathways.

To our knowledge, this study is the first report showing that EV71 infection activates JNK1/2 and p38 MAPK pathways in iDCs and leads to increased viral yield and proinflammatory cytokine secretions. Moreover, inhibition of JNK1/2 and p38 MAPK pathways could effectively reduces viral replication and cytokine release, supporting the idea that the activation of these two pathways are important for EV71 infection. We speculate that JNK1/2 and p38 MAPK regulate viral replication by acting at certain specific steps of viral replication cycle, including attachment, entry, gene transcription, protein expressions, and assembly, as well as viral pathogenesis. However, the underlying mechanisms need to be further studied in vitro or in vivo to highlight JNK1/2 or p38 MAPK as a potential broad antiviral molecular target for treatment of EV71 infection.

## Competing interest

The authors declare that they have no competing interest.

## Authors’ contributions

WS conceived and designed the experiments, participated in the data analysis and manuscript preparation. HP, LZ and LY performed cell culture, Western blot and flow cytometry. MS and YJ participated in the data analysis and manuscript preparation. JS and LZ performed PCR-fluorescence probing assay and analyzed the data. XW and XX detected cytokine levels. XZ and YM analyzed PCR array. All authors have read and approved the final manuscript.
